# Small non-coding RNAs, mammalian cells, and viruses: regulatory interactions?

**DOI:** 10.1186/1742-4690-4-74

**Published:** 2007-10-15

**Authors:** Man Lung Yeung, Monsef Benkirane, Kuan-Teh Jeang

**Affiliations:** 1 Molecular Virology Section, Laboratory of Molecular Microbiology National Institute of Allergy and Infectious Diseases, National Institutes of Health Bethesda, Maryland 20892-0460, USA; 2 Insitute de Genetique Humaine, Montpellier, France

## Abstract

Recent findings suggest that mammalian cells can use small non-coding RNAs (ncRNA) to regulate physiological viral infections. Here, we comment on several lines of evidence that support this concept. We discuss how viruses may in turn protect, suppress, evade, modulate, or adapt to the host cell's ncRNA regulatory schema.

## Small RNAs: interference and activation?

Plant and animal genomes have thousands of genes that encode non-protein-coding (nc) RNAs. While recent attention has focused significantly on small interfering RNAs (siRNAs) and micro RNAs (miRNAs), ncRNAs also include rRNA, tRNA, small nuclear (sn) RNA, small nucleolar (sno) RNAs, and some of the lesser-known RNAs such as vault RNAs, Y RNAs, rasi-RNAs and piRNAs [reviewed in [1]]. It is now recognized that only 2% of the human genome encodes for protein-coding RNAs while 60 to 70% of our DNA is transcribed into ncRNAs [2,3]. Hence, despite accumulating research on siRNA, miRNA and piRNA, we are likely at the tip of the iceberg in our understanding about functions and regulatory roles served by ncRNAs in cellular metabolism, pathogenesis and host-pathogen interaction.

**Figure 1:**
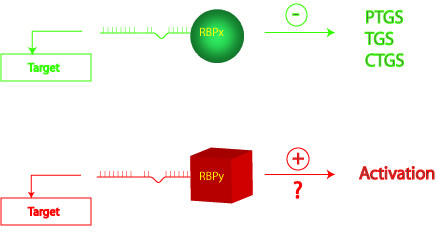
Schematic representations of positive and negative regulation mediated through ncRNA-guide sequences. RBPx is to illustrate a negative multi-RNA-binding protein regulatory complex that is tagged by a ncRNA-guide and recruited based on sequence-complementarity to target; while RBPy is to represent a theoretical positive multi-RNA-binding protein complex. PTGS, post-transcriptional gene silencing; TGS, transcriptional gene silencing; CTGS, co-transcriptional gene silencing. Currently, while there are many examples of RBPx, there is yet little published evidence for RBPy.

A key biological process served by small ncRNAs is a phenomenon termed RNA interference (RNAi). Recent reviews have reprised the discovery of RNAi and summarized the current state of knowledge about this process [4,5]. In brief, a central tenet of RNAi posits that small guide RNAs recruit, in a sequence-complementary manner, a multi-protein complex composed in part of RNA-binding proteins to RNA targets. This large multi-protein RNAi complex has been shown to include members of the Argonaute ribonuclease III protein family; and depending on biological context, the complex has been found to effect post-transcriptional gene silencing (PTGS), transcriptional gene silencing (TGS), and/or co-transcriptional gene silencing (CTGS) (Fig. 1, top).

Conventional wisdom suggests that biological processes are balanced by two principles, yin and yang, which oppose one another in their actions to confer equilibrium. For example, the cell-proliferative effects of oncogenes are countered by commensurate provocations of cellular senescence and apoptosis [6-8]. Moreover, frequently, potent transcriptional activators are also equally strong repressors [9,10]. Indeed, recent developments raise that the yin (negative) of RNAi may be shadowed by a yang (positive). Thus, some cellular [11-14] and viral [15] studies now suggest that a rarely-glimpsed face of "RNAi" may visualize activation rather than repression. Verily, RNA sequence-mediated positive regulation could exist more prevalently than currently acknowledged since, in principle, there is no reason why other RNA-binding proteins different from Argonaute-related members cannot be similarly tagged and guided by ncRNAs (Figure 1, bottom).

## Mammalian cells, viruses and small ncRNAs

Biological studies on mammalian viruses have illuminated aspects of gene regulation by small non-coding RNAs and their RNA-binding proteins. Early results from the HIV-1 TAR RNA and its binding protein Tat framed a platform for how a small non-coding RNA and a viral RNA-binding protein cooperate to up modulate gene expression [16,17]. Indeed, although unrecognized at the time, the first human cellular protein identified to bind TAR RNA, TRBP [18,19] presaged a clue to the mechanistic process of RNAi. Hence, fourteen years after the initial characterization of its binding to TAR, TRBP was revealed as a crucial component of the mammalian miRNA processing machinery [20-22]. Consistent with TRBP's role in miRNA-processing, a recent report demonstrated that TAR RNA in human cells is engaged by TRBP and processed by the RNAse III Dicer protein into a miRNA guide [23].

The above sequence of events not withstanding and although the RNAi machinery is preserved intact in mammalian cells and mammalian RNAi machinery can be instructed to target invading viruses in therapeutic settings, there is a school of thought that mammals do not use ncRNA/RNAi to regulate viral infections [24]. This view is partly rationalized by the argument that mammals have a surfeit of other means to defeat effectively viral pathologies; thus an intact mammalian RNAi machinery is not needed, and cells have extinguished this mechanism as it applies to viral infection [24]. Confoundingly, that annually 3 million human deaths arise from HIV-infection alone [25] and ~20% of all human cancers are caused by viral infections [26] indicate that mammalian defenses are not nearly so replete that effective antiviral pathway(s) should become extinct.

**Figure 2:**
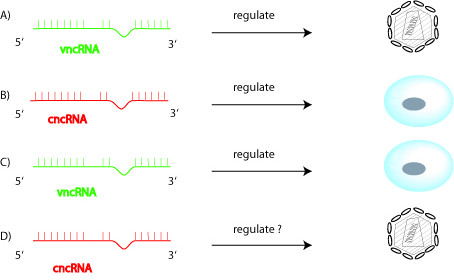
**Four different ways for viral (v) ncRNAs and cellular (c) ncRNAs to interact in mammalian cells.** A) shows vncRNA regulating virus; B) shows cncRNA regulating cells; C) shows vncRNA regulating cells; D) illustrates cncRNA regulating virus. Experimental evidence compatible with each of these four pathways exists in the published literature.

Recent experimental findings are, in fact, consistent with physiological use by mammalian cells of small ncRNA/RNAi to regulate viruses. First, three studies have converged to illustrate that small ncRNAs (siRNAs and piRNAs) are used in human and mouse cells to suppress the replication of endogenous retroviruses (i.e. retrotransposons) [27-29]. Second, bioinformatics and experimental results persuasively imply that mammalian viruses including HIV-1 are targeted by discrete human miRNAs [30-34]. Third, repression of mammalian Dicer enzyme was found to up regulate cellular replication of HIV-1 and vesicular stomatitis virus (VSV) [35-37]. One straightforward interpretation of the latter finding, which does not exclude others, is that the unrepressed mammalian Dicer-RNAi pathway normally acts to moderate HIV-1 and VSV replication. Finally, a KSHV viral miRNA (miR-K12-11) was identified as a viral orthologue of human miR-155 [38]. To the extent that miR-K12-11 targets KSHV- and cellular- sequences, then cellular miR-155 can be reasoned to act upon the same KSHV-sequence regulated by miR-K12-11. Indeed, pending the clarification of additional details, extant observations are consistent with the employment of ncRNAs by mammalian viruses to regulate viral functions, by mammalian cells to regulate cellular functions, by mammalian viruses to regulate cellular functions, and by mammalian cells to regulate viral functions (Figure 2).

## Viral responses?

If cells restrict viruses with non-coding RNAs, do viruses respond with countermeasures? In principle, viral counter stratagems could include a) protection from restriction, b) suppression of restriction; c) evasion from restriction; d) modulation of restriction profiles, and e) adaptation to restriction.

### Protection

Experimental findings suggest the existence for mammalian viruses of each of the above five mechanisms. Regarding protection, Berkhout and colleagues have reported that RNA genomes can be physiologically shielded from RNAi in a privileged format [39]. Nevertheless, this protection cannot prevail for unpackaged viral genomes or for transcribed viral mRNAs. Accordingly, viral mechanism(s) for RNAi-blunting or -suppression might be required in unprotected settings.

### Suppression

Indeed, viruses appear to possess at least two means to suppress RNAi. First, virus-encoded RNA-binding proteins partly serve an RNAi-neutralization function. This mechanism while well-accepted for viruses in lower eukaryotic cells [40] has been debated for mammals. Relevant to HIV-1, two studies have attributed RNAi-suppressive activity to the arginine-rich RNA-binding Tat protein [41,42], while a third study has questioned this concept [43]. Interestingly, the interpreted absence of Tat-associated RNAi-suppression in the latter study is complicated by high non-specific transcriptional activation of TAR-less cellular promoters by Tat, a finding inconsistent with the known specificity of Tat for TAR-RNA [44-46]. Second, in a separate way, mammalian viruses suppress RNAi by synthesizing small viral RNA decoys that competitively occupy RNAi machinery, preventing the processing of authentic RNAi precursors [47-49]. Because stringent global suppression of RNAi is likely incompatible with the viability of mammalian cells [50], virus-mediated RNAi-suppression is likely to be physiologically modest and localized in scope. This could explain difficulties in visualizing suppressive activities in non-viral transfection-based over expression assays.

### Evasion

**Figure 3:**
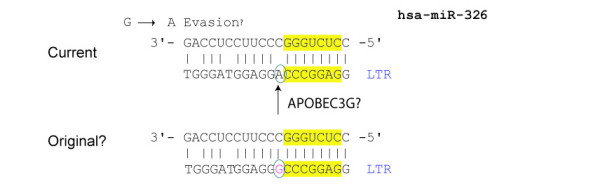
**A potential foot print of an HIV-1 escape mutation from human miRNA-mediated selection.** The HIV-1 sequence (bottom strand) shown in this figure is from the LTR of pNL4-3. Good base pairing of this sequence with human miR-326 (top strand) is shown (current); however, an even better base pairing (original?) is inferred if a putative APOBEC -mediated "G" to "A" change is corrected.

If protection or suppression fails, viruses can evade sequence-complementarity-driven RNAi through mutations. Highly mutable viruses like HIV-1 are proficient at sequence- or secondary structure- changes [51,52]. Experimental findings of rapid HIV-1 mutation under artificial siRNA-pressure do not, however, address whether ncRNA-based selection against HIV-1 exists physiologically in human cells. Here, supportive data are difficult to accumulate because by definition effective evasion (from e.g. miRNAs) means loss of evidence for base complementarity in viral genomes (to miRNAs). Hence, viruses with no human miRNA-footprints may actually be viruses that experience the strongest miRNA- selection. In a setting where an absence of finding may be indicative of evasion, how might one collect clues? An answer may reside with searching for human miRNA-target sites in viral genomes containing tell-tale mismatches. For example, rare HIV-1 sequence which appears to be targeted by human miRNAs (see Figure 3, an HIV pNL4-3 "target" of human miR-326) carries a hallmark change near the miR-seed sequence partly consistent with APOBEC (deoxycytidine deaminase) -mediated G to A mutation. In this example, one could change the putatively escaped "A" back to a "G" in the HIV genome, and test if the original "non-escaped" HIV-1 is subject to vigorous miR-326-selection. Such experimental design could provide findings supportive of a hypothesis that current HIV-1 RNA sequences are continually shaped and maintained by ambient RNAi pressure.

### Modulation and adaptation

Failing protection, suppression, and evasion from miRNAs, viruses may modulate the cell's miRNA expression profile [35,53], or viruses may ultimately adapt miRNA-restriction to benefit viral replication [15]. In the former setting, RNA-binding proteins such as Tat which act in the nucleus as well as affect cytoplasmic events via binding to tubulin [54-56] could significantly remodel cellular miRNA profiles [53]. In the latter scenario, human hepatitis C virus is currently the one rare example compatible with an adaptation paradigm [15]. As we learn more about miRNA -targets and -biology, better understandings of setting-specific negative versus positive modulations and adaptations will likely emerge.

## Perspectives and Predictions

We have outlined in brief two views on virus- cell ncRNA interaction in mammals. The first view embraces a null hypothesis --- virus-cell ncRNA interactions exist in lower eukaryotes but not physiologically in mammals [24]. While this view may be correct, several lines of evidence discussed here point against its limitations. A second view is that the RNAi machinery exists in mammalian cells not just for artificial siRNA-exploits but as a physiological mechanism used by cells and viruses to regulate viral and cellular functions (Figure 2). Subsumed within this view is the thesis that RNAi is a part of the armamentarium used by mammalian cells to regulate, perhaps positively and negatively in context-specific fashion, the replication of endogenous and exogenous viruses. We anticipate that more time and further investigation will be needed to validate the accuracy of the one or the other view point.

If small ncRNAs are used in mammalian cells to regulate cellular and viral functions, then one could venture several predictions. First, many more (cellular and viral) RNA-binding proteins that adapt small RNAs to mediate both negative and positive gene regulation will be revealed. Second, mammalian viruses will be shown to encode a variety of ncRNAs that have regulatory roles. Some future examples might mirror the HTLV-1 regulatory HBZ ncRNA [57]; others may emerge from the processing of antisense HIV-1 and HTLV-1 transcripts [58-60]; and even others may behave like TAR or RRE. Third, additional viral RNA-binding proteins (perhaps Rev and Rex) will be shown to have setting-specific RNAi- modulatory properties, and many viruses will be found to extensively reshape cellular miRNA expression profiles.

Since its first description a relatively short period of time ago, 9868 papers have already been published on RNAi (data from Pubmed search using the term, RNAi). An additional prediction (which will almost certainly be correct) is that RNA-guided gene regulation will continue to hold many exciting and unexpected scientific findings which will be published profusely in the coming years.
